# Gelling Power Alteration on Kappa-Carrageenan Dispersion through Esterification Method with Different Fatty Acid Saturation

**DOI:** 10.3390/gels8110752

**Published:** 2022-11-21

**Authors:** Yoga W. Wardhana, Nuur Aanisah, Iyan Sopyan, Rini Hendriani, Anis Y. Chaerunisaa

**Affiliations:** 1 Department of Pharmaceutics and Pharmaceutical Technology, Faculty of Pharmacy, Universitas Padjadjaran, Sumedang 45363, Indonesia; 2 Dosage Form Development Research Center, Faculty of Pharmacy, Universitas Padjadjaran, Sumedang 45363, Indonesia; 3 Department of Pharmacy, Faculty of Mathematics and Natural Sciences, Tadulako University, Palu 94118, Indonesia; 4 Department of Pharmacology and Clinical Pharmacy, Faculty of Pharmacy, Universitas Padjadjaran, Sumedang 45363, Indonesia

**Keywords:** gelling power, κ-carrageenan, esterification, fatty acids

## Abstract

The physicochemical properties of κ-carrageenan gels and their ester forms derived from different fatty-acid saturations were characterized and compared with those of native κ-carrageenan. Furthermore, stearic and oleic acids were used as the saturated and unsaturated fatty acids, respectively. Fourier-transform infrared (FTIR) spectra confirmed the introduction of the ester into the κ-carrageenan backbone. The thermogravimetric analysis showed that thermal stability increased along with the level of unsaturation, but there was a decrease in viscosity, hardness, and syneresis, which caused the consistency of the product to become more elastic. The results also showed that the ester form still has a swelling ability that is almost the same as that of κ-carrageenan. After being formulated into a gel dosage form, the product was successfully produced from the ester with unsaturated fatty acids, and it was more elastic than native κ-carrageenan and had good physical properties with spreadability that meets the requirements for topical preparations.

## 1. Introduction

κ-carrageenan is a hydrophilic linear sulfated galactan with a repeating polymer of disaccharide units extracted from red seaweed (*Eucheuma cottonii*) [1]. Furthermore, its ability to form a gel makes it a good gelling agent [2,3], thickener [4], and stabilizer [5] in various industries, such as the food, pharmaceutical, and cosmetic industries. There are three types of carrageenan with different viscosities, and they are classified based on the presence of a 3,6-anhydro galactose unit, as well as by the position of the sulfate group, namely kappa-carrageenan (κ-carrageenan), iota-carrageenan (ι-carrageenan) and lambda-carrageenan (λ-carrageenan) [6,7,8]. κ-carrageenan contains 25% sulfate ester and 34% 3,6-anhydrogalactose [9]. It also has the highest gelation strength compared to other types of polymers due to its constituents and helical structure [10,11]. 

In the development of gel dosage forms, it is necessary to have a base material with adjustable viscosity and elastic consistency so that it is easier to apply [12,13,14,15]. However, κ-carrageenan shows a rigid and dense consistency. Aqueous carrageenan solutions exhibit pseudoplastic behavior [16]. The sulfate groups in k-carrageenan cause repulsion between negative charges along the polymer chain, lowering the gelation power of k-carrageenan, and causing the shear-thinning response and high apparent viscosity to be detected, even at low shear rates (1 s^−1^). The mechanical properties of k-carrageenan gels are influenced by temperature [12]; concentration, type, and amount of metal salts [17,18]; and the presence of food ingredients such as sugars [19]. In order for k-carrageenan to be used as a stable gelling agent, and to obtain a suitable viscosity for topical preparations, the viscosity of carrageenan has been modified. Several studies have explored the modification and characterization of κ-carrageenan raw materials, such as esterification using fatty acids to replace some sulfates in its chemical structure. The results showed an alteration in bond mobility, which affected its gelling power [20].

In this study, the formation of κ-carrageenan esters was carried out through reactions with fatty acids using a catalyst with the reflux method. The fatty acids used included stearic and oleic acids, which are different from those in previous studies, namely decanoyl chloride, with a total of 10 carbon atoms [20]. These two compounds were used to compare the presence of unsaturated and saturated fatty acids in the esterification of κ-carrageenan. This was based on the different types of bonds between them. Oleic acid has 18 carbon atoms with one double bond, while stearic acid has 18 carbon atoms but no double bonds. The presence of different chemical bonds also affects the internal properties of a compound. After the formation of the κ-carrageenan ester, they were characterized to analyze the physicochemical properties of the gel.

## 2. Results and Discussion

### 2.1. Fabrication of Ester ĸ-Carrageenan

Esterification of kappa-carrageenan was accomplished by heating the polymer and the fatty acids in pyridine (as the catalyst) at 80 °C. The pyridine acted as both a solvent and a catalyst due to its success rate in the ester formation of several biopolymers [21,22,23]. It was also selected because it has electronegative nitrogen in the pyridine ring, which makes the molecule relatively electron deficient. Therefore, it is easier for the compound to enter into electrophilic aromatic substitution reactions. The choice of pyridine was also based on its pH value, with that being 8.45 because κ-carrageenan is hydrolyzed when dissolved in an acidic solvent.

The ratio of moles of κ-carrageenan to the fatty acids used was 1:6. This comparison is the optimal ratio, which was used to ensure that the fatty acids would be sufficient to react with κ-carrageenan. Reflux was carried out at 80 °C for 6 h, and this is the optimal condition for esterification because the product is often degraded at a temperature of >85 °C [20]. The hydrolysis reaction at high temperature, which reduces the polysaccharide chain’s molecular weight, led to the breakdown and breaking of the chain [24]. Although several studies have indicated that using higher temperatures can obtain a comparatively higher yield, the sulfate group in the carrageenan ester likewise disappears with an increase in temperature [25].

### 2.2. Characterization of Physicochemical Properties

#### 2.2.1. FTIR and TGA Analysis

**Figure 1:**
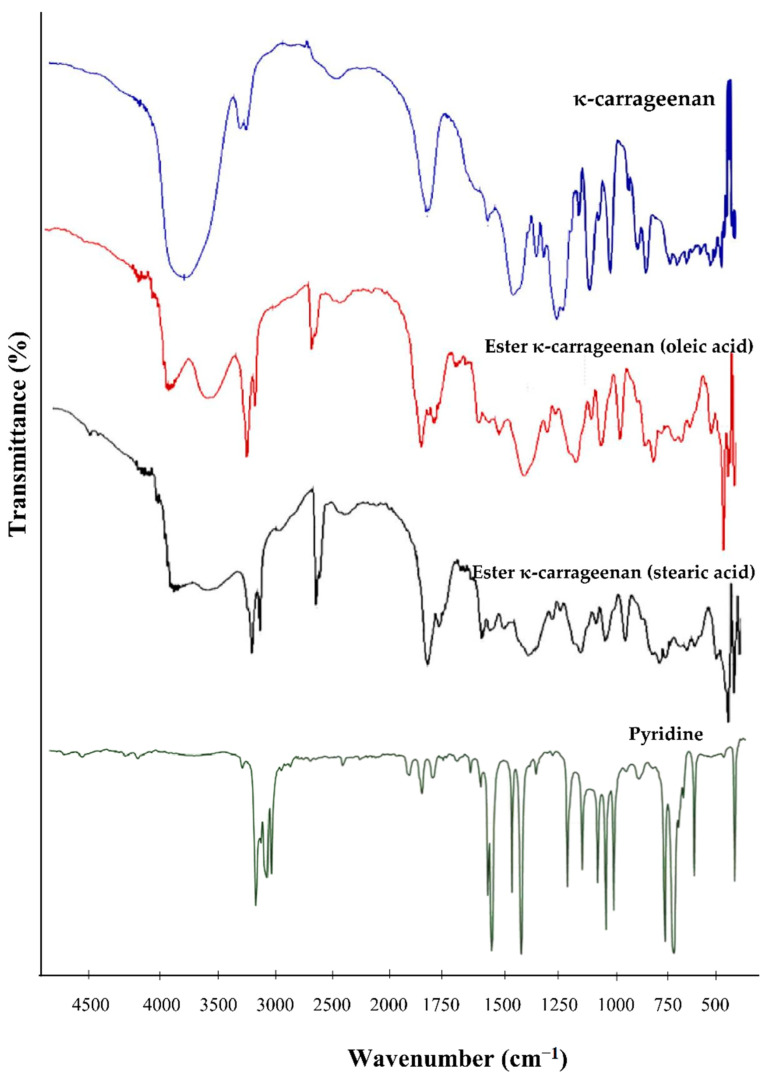
FTIR spectra of pyridine, κ-carrageenan, and its ester derivatives.

Figure 1 describes the FTIR spectra of κ-carrageenan and κ-carrageenan esters (oleic acid and stearic acid). The specific functional groups of κ-carrageenan in the FTIR spectrum are summarized as follows: 3000–3600 (O–H stretching), 1650 cm^−1^ (C–H bending), 1270–1250 cm^−1^ (O=S=O symmetric vibration), 928 cm^−1^ (C–O–C of 3,6-anhydrogalactose κ-carrageenan), and 845.80 cm^−1^ (O–SO_3_ stretching vibration). This is in accordance with the research of Mahmood, et al. (2014), who reported that the wavenumbers mentioned are characteristic of κ-carrageenan [20]. κ-carrageenan esters show that the peak area of the –OH group is not as broad as the peak of the –OH groups shown by κ-carrageenan. It can be seen in the Figure 1 that the peak area of the –OH group of κ-carrageenan esters is not as strong when compared to κ-carrageenan. This indicates that there is a reduction in the –OH group during the esterification process, that there is a substitution of acyl groups in κ-carrageenan, and that there is the formation of an ester. The esterification process can also be confirmed by looking at the spectrum of the κ-carrageenan ester (with oleic acid and stearic acid), which shows the presence of a carbonyl ester peak at 1706–1708 cm^−1^, which was previously not seen in κ-carrageenan. The result confirms the introduction of the ester into the κ-carrageenan backbone. In our study, the removal of pyridine in the κ-carrageenan ester was carried out using the simplest method, that being the evaporation of trace amounts of pyridine in the product at a temperature of 60–80 °C until the smell of pyridine vanishes. This is confirmed by the absence of a pyridine IR spectrum at a wavelength of 1455 cm^−1^ [26]. Thus, ester derivatives of κ-carrageenan are free of pyridine, and pyridine acts only as a catalyst. Then, to remove unreacted fatty acids effectively, we washed the product with ethanol before drying.

**Figure 2:**
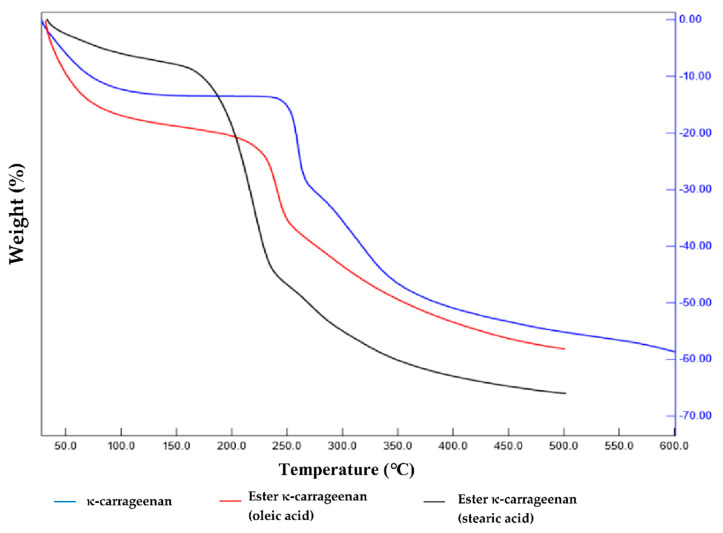
TGA thermogram of κ-carrageenan and its ester derivatives.

Thermal gravimetric analysis was carried out to evaluate the thermal stability of κ-carrageenan and its ester derivatives, as shown in Figure 2. The study of the thermal stability of polymers is very important in the design of their products to determine the temperature range of the material and ensure that it can be used without degradation. The weight reduction in polymers and the development of hydrogels occur in three stages. In the first stage, a weight reduction was perceived by the TGA thermogram of κ-carrageenan and its ester derivatives within the temperature range of 30–100 °C due to the moisture loss as polysaccharides usually have a strong affinity for water. κ-carrageenan undergoes a weight loss at higher temperatures than do the ester derivatives due to the presence of a hydroxy structure in the anhydrogalactose carrageenan. It also contains hydrogen bonding between hydrogen and oxygen atoms, which is one of the strongest types of chemical bonds, thereby making it difficult to decompose. The carbonyl bond (C=O) between the ester chain and the hydroxyl group of the anhydrogalactose unit has weaker molecular interactions; hence, it disintegrates more easily at lower temperatures [27].

In the second stage, a further weight reduction was detected as the temperature reached 170–350 °C, and this can be attributed to the primary degradation of the sulfonate groups of the polymer [28,29]. The ester (stearic acid) underwent the most rapid decomposition, with a peak at 178 °C. Meanwhile, the second ester (oleic acid) and κ-carrageenan underwent decomposition with peaks at 234 °C and 254 °C, respectively. This stage is usually referred to as the devolatilization stage, where the main pyrolytic process occurs and various volatile components can be released gradually.

The third stage of decomposition occurred at temperatures above 350 °C, and κ-carrageenan stearate started to decompose rapidly around 340 °C. This step can be attributed to the residue from the previous pyrolytic process, which slowly decomposed into a loose, porous residue. By comparing the decrease in weight among all of the ester derivatives, it can be seen that oleic acid has a level of thermal stability similar to that of κ-carrageenan.

#### 2.2.2. Physical Properties of κ-Carrageenan and Its Ester Derivatives

As a result of esterification, the product was obtained in the form of a coarse and odorless powder. The esterification of κ-carrageenan with oleic acid showed a yellowish-white color, while that with stearic acid showed a grayish-white color. This difference was influenced by the color of each fatty acid used. The result of κ-carrageenan ester (oleic acid) was yellowish because the oleic acid used was a yellow liquid; hence, the product obtained was yellowish-white. Meanwhile, the product obtained with stearic acid was a white powder. 

**Figure 3:**
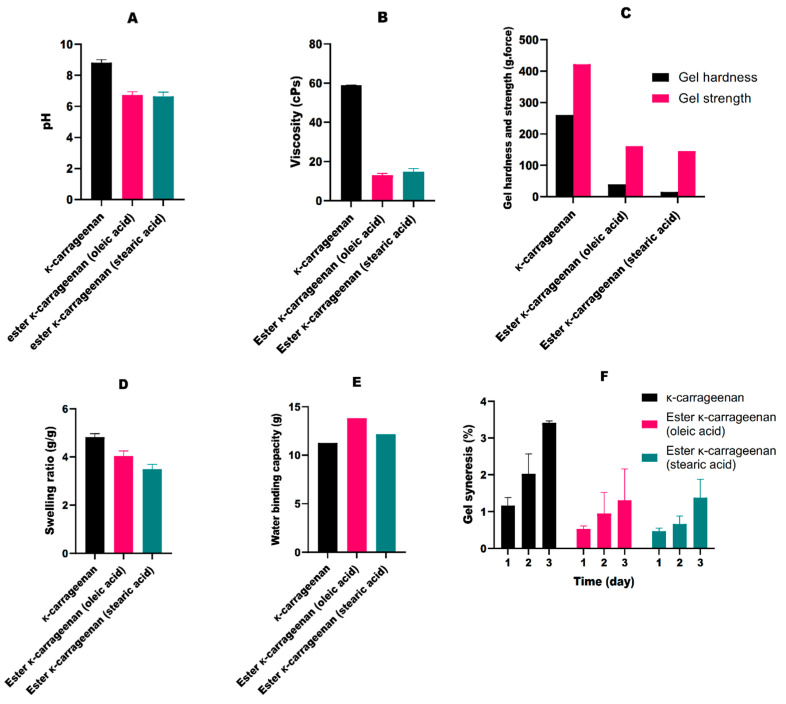
The pH (**A**), viscosity (**B**), gel hardness and strength (**C**), swelling ratio (**D**), water-binding capacity (**E**), and syneresis (**F**) of κ-carrageenan gel in comparison with its ester derivatives (means ± SD, n = 3).

Figure 3 shows the physical properties of κ-carrageenan and its ester derivatives (oleic acid and stearic acid) from fatty acids. The results of pH testing on the samples are presented in Figure 3A, where κ-carrageenan shows a higher pH value when compared to those of the esters. This occurs because the esterification reaction with the addition of fatty acids decreases the pH value of the products.

The test results for the viscosity of κ-carrageenan and κ-carrageenan esters can be seen in Figure 3B. The viscosity and rheology of kappa-carrageenan were measured at 60 °C because the kappa-carrageenan gel had a solid consistency at room temperature. This dense gel consistency is due to the low sulfate ester content of kappa-carrageenan. In addition, the sulfate group on carrageenan causes repulsion between the negative charges along the polymer chain, causing the molecular chain to stiffen [30]. However, after esterification, the kappa-carrageenan decreased in viscosity. The viscosity of the κ-carrageenan ester reduces significantly with an increase in unsaturation so that the esterification using oleic acid shows a lower viscosity, even with the same number of carbon atoms in the chain of fatty acids. It is known that oleic acid’s double bonds alter the conformation of the κ-carrageenan’s structure, causing tangling in its straight chain and preventing the structure from stacking closely together. Thus, a κ-carrageenan ester utilizing unsaturated fatty acids does not have a rigid and fixed structure, resulting in a reduction of the κ-carrageenan ester solution [31,32]. Then, the decrease in the viscosity of the κ-carrageenan ester can be caused by the breaking of hydrogen bonds and the weakening of the intermolecular forces of carrageenan after the introduction of the carbonyl group. In addition, the introduced branched chain can inhibit the chain linkage of the carrageenan molecules, resulting in a decrease in the viscosity of the κ-carrageenan ester. Furthermore, the partial hydrolysis of carrageenan chains under basic esterification can also decrease the viscosity [27].

The characteristics of non-Newtonian fluids are indicated by the high-molecular-weight hydrocolloids, including carrageenan. The results of rheological testing on all samples showed the presence of pseudoplastic properties. The flow property experienced a decrease in shear stress with increasing shear velocity, or it showed a decrease in viscosity with an increase in shear rate [16].

Gel hardness is defined as the maximum peak force at the first compression, while gel strength is the maximum force required to break the polymer matrix in the stressed area. Based on Figure 3C, the κ-carrageenan gel showed a higher strength value than those obtained from the esters. Rey et al. [33] showed that the increase in strength is directly proportional to 3,6-anhydrogalactose and inversely proportional to the sulfate content. The presence of 3,6-anhydrogalactose causes orderliness in the polymer and enhances the potential for double-helix formation. The esterification results showed a low gel strength value. This was due to the higher number of ester groups, which bind to water when compared to κ carrageenan. Yuguchi et al. revealed that the formation of gel in carrageenan is a deposition involving ionic bonds between certain metal cations and the negative charge of the sulfate ester group [7]. When the number of sulfate ester groups is greater, then the sulfate binds to water. Therefore, if the sulfate content in carrageenan is high, the three-dimensional framework that is formed tends to absorb a lot of water. This makes it difficult for the gel to maintain its shape under pressure, thereby leading to a low strength value.

κ-carrageenan gel has a high level of hardness due to its hard and rigid texture. The high hardness value occurred because carrageenan can lead to the formation of aggregates due to the presence of a double helix. As more double strands are formed, the crosslinks between the strands also produce large amounts of aggregate in the form of strong nets. This leads to smaller intermolecular spaces, and the free water in the gel is pushed out, thereby making it harder [34,35]. The esterification carried out on κ-carrageenan led to a decrease in the hardness of the product because its texture was getting softer. The gel produced from esterification can absorb large amounts of water, which cause an increase in the amount of free water, as well as a softer texture.

The swelling characteristics of hydrogels are one of the important parameters in biomedical and pharmaceutical applications because the equilibrium swelling ratio affects the solute diffusion coefficient, surface wettability, and mobility, as well as optical and mechanical properties. Figure 3D shows that κ-carrageenan and esterified gels have similar ratios. The swelling rate in the three samples occurred due to the presence of a more rigid bonding strength [36]. They also have the ability to expand, and their levels of elasticity are very similar. Gels can expand because the gelling components can absorb the solution, which leads to an increase in volume. The solution also penetrates the gel matrix, and this causes interactions between the solution and the gel. 

“Water-binding capacity” describes the ability of a material to bind water and the tendency of water to associate with hydrophilic substances. κ-carrageenan has a high capacity due to its hydrophilic sugar structure, as well as the amorphous molecular arrangement of the polysaccharide chains [37]. It is also related to the sulfate group, which is very polar in its structure [38]. Figure 3E shows that the water-binding capacities of the esters were greater than that of κ-carrageenan. 

The results of the syneresis test of κ-carrageenan and the esterification gel are presented in Figure 3F. Syneresis is the shrinkage of the product to squeeze out water from the gel, which makes it appears smaller and denser. Extreme cold temperatures cause the formation of aggregates, and these increase the strength of the gelling polymer. Therefore, the bound water comes out of the gel. The results show that the ester experienced lower syneresis compared to the κ-carrageenan. The rate for the gel was good when the percentage was not more than 1%. Based on the test results, κ-carrageenan ester gels can be classified as good based on the average value obtained over 3 days, which was less than 1%. This was due to the addition of an ester group that replaced the OH group in κ-carrageenan. The replacement then stretches the distance and bonding cavities between the polymers, thereby leading to the retention of more water, while Lee et al. (2008) stated that the number of bonding or junction zones is one of the causes of a high level of syneresis in κ-carrageenan. Therefore, the product is physically unstable and cannot store water at ±10 °C [39,40]. 

#### 2.2.3. Physical Stability and Properties of Optimized Gel Base

Optimization of the gel base concentration was carried out by preparing a gel base using κ-carrageenan and its esters with a concentration variation of 0.3–2% with distilled water. Furthermore, the stability of the product was then evaluated for 1 week. The characteristics tested included viscosity, pH, dispersion, homogeneity, and organoleptic properties, such as odor, color, and consistency. One of the concentrations of κ-carrageenan and its ester, which has a level of stability that meets the requirements or standards, was selected.

Based on the results of our organoleptic observations, κ-carrageenan, ester (oleic acid), and second ester (stearic acid) bases with concentrations of 0.6%, 0.8%, and 1.6%, respectively, did not change, and the color obtained was clear. For the organoleptic observation of odor, only the κ-carrageenan gel base gave off a fishy smell. The product obtained from κ-carrageenan produced a rigid gel after 1 week of observation, while that of the esters was not rigid.

The homogeneity test showed that the gel base of κ-carrageenan and its ester (oleic acid) did not show any coarse grains when viewed visually, which indicates their homogeneity. Meanwhile, the product from the ester (stearic acid) gel base had coarse grains, which indicate that it is not homogeneous.

A physical stability test of the gel base was conducted over the course of 7 days for changes in pH, viscosity, and dispersion, as shown in Table 1.

The pH test was carried out to determine the compatibility of the ingredients with the skin. This is associated with the safety and comfort of the preparation during usage. If the pH is not consistent with that of the skin, the preparation can cause irritation, which leads to discomfort. Based on Table 1, there was an increase and a decrease in the pH value of the preparation. However, the change was insignificant, and the value obtained was still within the normal range, namely 5.0–6.8 [41].

Table 1 shows that κ-carrageenan and its esters experienced an increase in viscosity. κ-carrageenan is a stiff gel and is difficult to pour, while the esters have a fairly stable consistency. This was due to the fact that the increase was relatively insignificant over the 7 days of storage. κ-carrageenan is a polymer, and its molecules enter the cavity formed by water during dispersion. This leads to the formation of hydrogen bonds between the hydroxyl (OH) groups and water molecules. The bonds play a role in the hydration of the swelling process; hence, the higher the concentration of κ-carrageenan, the more hydroxyl groups that will bind, and the higher the viscosity will be.

The spreadability test was used to determine how well the gel preparation spreads on the skin surface because it can affect both the drug absorption and the speed of the release of the active substance when applied. A good preparation can spread easily on the skin and is comfortable to use. Based on the predetermined standards, a good level of spreadability falls within the range of 5–7 cm. The gel base that is closest to the requirement was obtained from the ester using oleic acid with a concentration of 0.8%. κ-carrageenan gel does not spread due to its rigidity, while that of the ester (stearic acid) can spread, but it does not have the required dispersion value.

Overall, the findings presented in this study indicate that the unsaturation of the double bonds in fatty acids affects the physical properties of the κ-carrageenan ester gel formed, namely increasing thermal stability as well as decreasing viscosity and syneresis. After being formulated into the gel in a dosage form that can be applied to the skin, the ester using an unsaturated fatty acid provided organoleptic properties, spreadability, and pH.

## 3. Conclusions

κ-carrageenan esters were synthesized from κ-carrageenan using unsaturated and saturated fatty acids, namely oleic acid and stearic acid, respectively. The effect and physicochemical properties of the gel formed were then assessed. TGA results showed that the esters from unsaturated fatty acid (oleic acid) are more thermally stable and increase the water-binding capacity, which makes the product more elastic. The addition of ester groups using unsaturated fatty acids also lowers the viscosity and syneresis due to the unsaturation from the spatial configuration of the double bonds impairing the efficiency of the crystal packing. The results also revealed that the gels produced were more easily dispersed. All the data obtained represent new evidence that the chemical modification of κ-carrageenan to its ester form using an unsaturated fatty acid produces a gel with the desired physical properties, which can be considered for use in pharmaceutical applications.

## 4. Materials and Methods

### 4.1. Synthesis of Ester ĸ-Carrageenan

Kappa-carrageenan (ĸ-carrageenan) is a local product that has been widely marketed. It was obtained from CV Nurajaya, a supplier in Surabaya, Indonesia. The ĸ-ester was prepared by adding 35 mL of pyridine to 0.007 mol of ĸ-carrageenan at 80 °C under vigorous stirring for 30 min. It was then mixed with 0.05 mol of oleic acid or stearic acid and refluxed at 80 °C for 6 h. The reaction mixture obtained was left at room temperature for precipitation and washed with ethanol. The precipitate was then dried in an oven at 80 °C, and it formed a thin layer, which was further crushed into small particles and washed with ethanol, followed by drying at room temperature for 24 h [20].

### 4.2. Characterization of Physicochemical Properties

#### 4.2.1. Fourier-Transform Infrared Spectroscopy (FTIR)

The functional groups of the samples were analyzed by Fourier-transform infrared spectroscopy (IR Prestige 21). A sample of 0.02 g was mixed with KBr powder and pressed to form a thin film. Spectral identifications were performed in the wavenumber range 600–4000 cm^−1^ with four scans at a resolution of 0.4 cm^−1^. 

#### 4.2.2. Thermogravimetric Analysis (TGA)

A quantity of 2 mg of unesterified and esterified ĸ-carrageenan was placed in an alumina crucible on the TG/DTA sample kit and subjected to thermogravimetric analysis (TGA) using a HITACHI STA7300. Then, the sample was heated from 30 to 500 °C at a constant rate of 10 °C/min under a nitrogen atmosphere. The derivative curve of thermogravimetric analysis, differential thermogravimetric (DTG), was obtained from the derivative of the first-order curve.

#### 4.2.3. Organoleptic Assessment

Organoleptic assessment of kappa-carrageenan powder, whether esterified or not, includes the direct examination of the shape, color, and odor.

#### 4.2.4. pH

The apparent pH of the carrageenan sample, with a concentration of 1.5% in hot, distilled water, was measured using a digital pH meter under 25 ± 1 °C.

#### 4.2.5. Viscosity

Briefly, the 1.5% *w*/*v* unesterified and esterified ĸ-carrageenan samples were dispersed in hot, distilled water up to 50–100 mL. The viscosity measurement was carried out to determine the level of gel viscosity at a certain concentration using a Brookfield DV-E Series Viscometer with a spindle number 1 at a speed of 60 rpm and at 25 ± 1 °C. The optimum viscosity of the gel preparation is 2000 cPs [42].

#### 4.2.6. Rheological Properties

The type of flow was determined by measuring the rheological properties using a viscometer (Brookfield Engineering Labs Inc., Middleboro, MA, USA). The gel was placed into the sample container; the spindle was rotated from a low rotation speed to a high speed; then, the reverse was carried out; and the torque value obtained was 10–100%. A rheological graph was developed by entering data on viscosity and speed (rpm), after which it was input into an equation:

(1) $$shear\;rate\;\left(sec^{-1}\right)=viscosity\;\left(poise\right)\times 7.187$$

(2) $$shear\;force\left(\frac{dynes}{{cm}^{2}}\right)=\frac{1}{visosity}\times shear\;rate$$

#### 4.2.7. Gel Strength and Hardness

A texture analyzer (Stable Micro System TA-XT, Surrey, UK) was used to determine the gel strength produced from several variations of carrageenan concentrations. Furthermore, gel strength is the maximum force required to break the polymer matrix in the compressed area. The sample produced was inserted into a container, and the penetration device was lowered to the surface of the product. It was then measured when the plunger had penetrated 4 mm. Gel strength (g mm) was calculated by multiplying the penetration force (g) by with distance of the penetration (mm). A P/1KSS probe was used with a length of 4 mm and a speed of 0.5 mm/second. For the hardness test, an SMS P/6 probe was used with a trigger force of 1 g, a distance of 5 mm, and a crosshead speed of 5 mm/s. Hardness, cohesiveness, springiness, chewiness, gumminess, and adhesiveness were calculated from the force-time curves.

#### 4.2.8. Swelling Ratio

The swelling ratio test was carried out to calculate the hydrogel sorption capacity, which can be determined by the teabag method using distilled water at room temperature (Zhao, 2005) [43]. The gel mixture was made and then placed in a Petri dish. The gel was dried at 40 °C for 2 days until the gel weight was constant. The teabags used were non-woven bags. Empty, non-woven bags were weighed as *W_n_*, and the weight of the dry gelling agent was weighed as *W_o_*. The non-woven bag was filled with a dry gelling agent mixture, soaked in distilled water, and checked for weight every 10 min until the weight was constant. Then, the non-woven bag was lifted, hung up, and allowed to drip after it was weighed as *W_s_*. The water absorbed by the gelling agent can be calculated using the formula (3):

(3) $$Swelling\;ratio=\frac{Ws-Wo-Wn}{Wo}\times 100\%$$

where *Wn* is the weight of the empty teabag; *Wo* is the weight of the dried gelling agent; and *Ws* is the weight of the hydrogel when expanding.

#### 4.2.9. Water-Binding Capacity

The sample was mixed with distilled water to form a gel, after which it was then weighed. The gel was transferred to a tube and centrifuged for 2 min, and it was allowed to stand for 30 min at 25 °C. The supernatant was discarded, and the weight gain was calculated as the binding capacity [44].

#### 4.2.10. Gel Syneresis

Syneresis is the release of water from the gel due to shrinking, thereby making it denser. Furthermore, the parameter was observed by weighing 70 g of gel in a clear pot and leaving it for 3 h to be equilibrated at room temperature. The samples were then incubated at the refrigerated temperature of 2–8 °C for 24, 48, and 72 h, followed by centrifugation at 2150× *g* at 25 °C for 10 min to separate the water and the gel. The syneresis was calculated by measuring the weight of the gel after discarding the separated water, as compared to the initial weight, which can be expressed by the following formula [45,46]:

(4) $$Gel\;syneresis\;\left(\%\right)=\frac{Initial\;weight-Final\;weight}{Initial\;weight}\times 100\%$$

The syneresis rate for the gel is good when the syneresis percentage is not more than 1% [47].

## Figures and Tables

**Table 1:** Physical stability of the optimized formula during storage (7 days).

Parameter	F1				F2				F3			
	1	3	5	7	1	3	5	7	1	3	5	7
pH	7.28	7.29	7.34	7.34	4.7	5.37	5.4	5.31	5.72	5.95	6.09	5.98
Viscosity (cPs)	8.6	9.88	9.99	9.99	2.33	2.43	2.55	2.66	3.77	3.85	4.27	4.54
Spreadability (cm)	3	3.4	3.4	3.5	5	5.2	5.41	5.53	3.77	3.85	4.27	4.54

## Data Availability

Not applicable.
